# Open Biology in a new decade

**DOI:** 10.1098/rsob.200025

**Published:** 2020-02-05

**Authors:** David M. Glover

**Affiliations:** Editor-in-Chief

The end of the decade marks the end of my tenure as Editor-in-Chief of Open Biology. How far have we come since the launch of the journal in September 2011? This has certainly been a difficult time to start a new journal in molecular and cell biology, where the competition is fierce. We have made impact, however, with our dreaded Impact Factor oscillating between 3.5 and 5.8 during this time. I anticipate the metrics will soon rise higher because, most importantly, we are now recognized within the community as a journal that truly represents the interests of scientists. There has been a lot of debate about scientific publishing in the past decade and we have seen many new models emerge. We have watched this happen and taken up many suggestions from our readers and editorial board. While considering many different options, however, we have continued to follow a fairly traditional pattern of peer review. I believe this works well for us exactly because the journal is run by scientists for scientists and we know of the problems facing scientific publishing, particularly in molecular and cell biology, and wish to avoid them. It is vital that the academic academies and societies provide their members with publishing opportunities and indeed, the Royal Society serves us all well in this respect. Whereas competition from the publishing houses keeps us on our toes, our motivation is simply one of scientific communication to aid scientific progress.

In trying to broaden our ability to communicate, I have had a particular focus over the past 3 or 4 years. This is to promote the publication of more review articles in the journal. I see this as an important role for the journal and I am delighted that I will be maintaining my connection with the journal as Reviews Editor from 2020. I am very pleased to welcome Jon Pines as my successor as Editor-in-Chief. I am certain that Jon will bring new ideas for steering the journal to greater success.

During my own tenure as Editor-in-Chief, I have had tremendous help and assistance from many of the staff in Royal Society publishing. Of these, I would like to single out Buchi Okereafor, in particular, for her unfailing help and cheerful optimism. I am also grateful for the help of my wonderfully organized secretary Kseniya Tyshkevych that has enabled us to write emails to literally thousands of scientists all over the world and so make direct contact with the community.

A new decade brings new challenges and opportunities for all of us. I am now spending much more of my time in Pasadena than Cambridge and have a delightful new set of colleagues. The journal is in new hands and yet has the continued support of many of its original editorial team. I am therefore confident that it will continue to make new inroads representing the academic society that was first to establish a scientific journal over 350 years ago. We continue to build for the next 350 years and beyond.

## Supplementary Material

Reviewer comments

