# Supplementary material for: Open Biology in a new decade
Source: Open Biol. 2020 Feb 5;10(2):200025. doi: 10.1098/rsob.200025 (PMC7058933; doi:10.1098/rsob.200025)
Supplement: Reviewer comments [file rsob200025_review_history.pdf]

THE ROYAL SOCIETY
PUBLISHING

OPEN
BIOLOGY

Open biology in a new decade

David M. Glover

Article citation details
Open Biol. **10**: 200025.
http://dx.doi.org/10.1098/rsob.200025

Review timeline
Original submission: 22 January 2020
Final acceptance: 22 January 2020

Note: Reports are unedited and appear as submitted by the referee. The review history appears in chronological order.

Review History

Decision letter (RSOB-20-0025.R0)

22-Jan-2020

Dear Professor Glover

We are pleased to inform you that your manuscript entitled "Open Biology in a New Decade" has been accepted by the Editor for publication in Open Biology.

You can expect to receive a proof of your article from our Production office in due course, please check your spam filter if you do not receive it within the next 10 working days. Please let us know if you are likely to be away from e-mail contact during this time.

Thank you for your fine contribution. On behalf of the Editors of Open Biology, we look forward to your continued contributions to the journal.

Sincerely,

The Open Biology Team
mailto: openbiology@royalsociety.org

Reports © 2020 The Reviewers; Decision Letters © 2020 The Reviewers and Editors; Responses © 2020 The Reviewers, Editors and Authors. Published by the Royal Society under the terms of the Creative Commons Attribution License http://creativecommons.org/licenses/by/4.0/, which permits unrestricted use, provided the original author and source are credited
